# Editorial for the Special Issue on Advances in Low-Dimensional Materials: Synthesis, Characterization, and Device Applications

**DOI:** 10.3390/mi16060693

**Published:** 2025-06-10

**Authors:** Peixun Wu, Xiaotian Zhang

**Affiliations:** School of Mechanical Engineering, Shanghai Jiao Tong University, Shanghai 200240, China

Low-dimensional materials, encompassing two-dimensional (2D) layers, one-dimensional (1D) nanowires, and zero-dimensional (0D) quantum dots, have revolutionized materials science over the past two decades. Their unique electronic, optical, mechanical, and thermal properties—arising from quantum confinement effects and high surface-to-volume ratios—have unlocked transformative applications in electronics [1], energy storage [2,3], sensing [4], and biomedicine [5]. These materials exhibit remarkable versatility, enabling advancements in energy-efficient transistors [6], flexible electronic devices [7], highly sensitive sensors [8], and next-generation batteries [9]. Furthermore, their quantum-scale phenomena have opened up new possibilities in photonics [10], quantum computing [11], and environmental remediation [12], driving innovation across diverse scientific domains. This Special Issue, comprising six original research articles and two review papers, brings together some of the latest research addressing the challenges and discoveries in synthesizing these materials, developing characterization techniques, and integrating them into functional devices. By bridging the gap between fundamental research and real-world applications, this issue aims to spark the development of low-dimensional materials for emerging technologies, fostering interdisciplinary collaboration and paving the way for breakthroughs in nanotechnology and beyond.

For example, in terms of flexible sensing applications, Zhao et al. synthesized an elastic macroporous graphene aerogel (MGA) using a modified Hummers method and assembled a flexible MGA-ETPU sensor by combining MGA with expanded thermoplastic polyurethane (ETPU) particles [13]. Compared to existing flexible sensors, the MGA-ETPU sensor exhibits superior performance, featuring excellent conductivity, exceptional elasticity, simple fabrication, high sensitivity, rapid response, and robust flexibility—making it ideal for flexible pressure sensing in smart footwear.

In terms of photonic applications, Jang et al. investigated inverted red quantum dot light-emitting diodes (QLEDs) using ZnO nanoparticles synthesized in open and closed systems [14]. Wurtzite-structured ZnO nanoparticles were prepared from zinc acetate dihydrate and potassium hydroxide at varying temperatures, with the study systematically analyzing how synthesis environments impact particle size and QLED performance. Key findings reveal the solvent evaporation’s critical role in nanoparticle growth. This work provides a novel strategy for enhancing inverted QLED performance without complex structural modifications.

What is more interesting, Jasulaneca et al. designed a single-transistor preamplifier circuit with Bi2Se3 nanowires for the electrical detection of mechanical oscillations in nanoelectromechanical systems (NEMSs) at cryogenic temperatures [15]. Integrated near the nanowire within a cryostat to minimize cable loading and interference, the circuit acts as an impedance converter for high-internal-resistance NEMS current measurements. Additionally, by placing the preamplifier near the resonator, parasitic wire capacitance contributions are minimized, enabling straightforward readout at the excitation frequency. This design provides a foundation for precise NEMS-based switching devices operating at cryogenic temperatures, relevant to quantum and space technologies.

In terms of energy and the environmental perspective, Reddy et al. employed a simple and cost-effective approach to enhance the photoelectrochemical (PEC) water-splitting performance of various materials, including reduced graphene oxide (rGO), tin oxide nanostructures (SnO_2_), and rGO/SnO_2_ composites [16]. This study utilizes low-cost microwave reduction and solution-based methods to prepare high-performance low-dimensional heterostructures. These nanostructures can be applied to both PEC water splitting and other photoelectrocatalytic processes such as CO_2_ reduction, pollutant degradation, and energy storage systems like supercapacitors and batteries.

In the next study, Honciuc and Negru reported a simple, equipment-free method for fabricating Janus films via Pickering emulsion interfacial self-assembly and polymerization [17]. The asymmetric nanostructures enable multifunctional wettability control, where hydrophilic/hydrophobic or superhydrophobic properties can be flexibly tailored through nanoparticle surface chemistry and hydrophobic treatments. By adjusting the wettability contrast across the film’s two sides, these Janus films hold significant potential for applications ranging from water distillation and desalination to emulsion stabilization and biomedical membrane fabrication.

Rabia et al. synthesized Ag_2_S-Ag_2_O-Ag/poly(2-aminobenzene-1-thiol) (P2ABT) nanocomposites via photopolymerization using AgNO3 as an oxidizer [18]. The nanocomposites were tested in acidic and alkaline media, demonstrating superior performance in supercapacitor applications. Notably, the specific capacitance, energy density, charge transfer resistance, and stability were significantly enhanced in acidic media compared to alkaline conditions.

In addition, we have two comprehensive reviews in the Special Issue regarding the State-of-the-Art in mechanical ultraprecision machining for nano/micro device manufacturing and recent advances in low-dimensional carbon dots (CDs) for environmental applications. The first review systematically analyzes the application of mechanical ultraprecision machining in brittle-hard materials, elucidates plastic domain processing mechanisms (e.g., high-pressure phase transitions, dislocation motion), evaluates advanced techniques (e.g., laser-assisted, ultrasonic vibration machining), and emphasizes sustainable manufacturing [19]. By integrating experimental and molecular dynamics approaches, the review provides a clear roadmap for advancing high-precision, low-environmental-impact nano/microfabrication technologies.

The second review summarizes the design of ratiometric fluorescent probes utilizing carbon dots (CDs) and their applications in environmental monitoring [20]. The discussion encompasses construction strategies, the principles of ratiometric fluorescence, and their implementation in detecting diverse environmental pollutants, including organic contaminants, heavy metal ions, and other substances. The review further explores the associated advantages and challenges, proposes potential solutions, and outlines future research directions.

The collective works in this Special Issue exemplify the transformative potential of low-dimensional materials, bridging atomic-scale precision with macroscale functionality. From flexible sensors and quantum-enabled NEMSs to sustainable energy solutions and environmental remediation, the featured studies underscore the synergy among synthesis, characterization, and applications. We extend our gratitude to the authors, reviewers, and editorial team for their contributions to this collection. As the field evolves, interdisciplinary collaboration will remain key to unlocking technological frontiers, addressing global challenges, and redefining nanotechnology’s future.
